# Supplementary material for: N-glycan-modified α-L-iduronidase produced by transgenic silkworms ameliorates clinical signs in a Japanese macaque with mucopolysaccharidosis I
Source: Commun Med (Lond). 2025 Apr 18;5:128. doi: 10.1038/s43856-025-00841-7 (PMC12008302; doi:10.1038/s43856-025-00841-7)
Supplement: Supplementary file 2 — Supplementary Information [file 43856_2025_841_MOESM2_ESM.pdf]

Supplementary information

N-glycan-modified α-L-iduronidase produced by transgenic silkworms ameliorates clinical findings in a Japanese macaque with mucopolysaccharidosis I

Chika Shinoda[1#], Keisuke Kitakaze[2#], Yuya Sasai[1], So-Ichiro Nishioka[1], Isao Kobayashi[3], Megumi Sumitani[3], Ken-Ichiro Tatematsu[3], Tetsuya Iizuka[3], Akira Harazono[4], Ai Mitani[5], Akihisa Kaneko[6], Masanori Imamura[6,7], Takako Miyabe-Nishiwaki[6], Yasuhiro Go[8,9], Akihiro Hirata[10], Yoshie Takeuchi[11], Teru Mizuno[1], Kei Kiriyama[1], Jun Tsukimoto[1], Satomi Nadanaka[12], Akiko Ishii-Watabe[4], Takashi Kinoshita[5], Hiroshi Kitagawa[12], Yasuyuki Suzuki[13], Takao Oishi[6*], Hideki Sezutsu[3], Kohji Itoh[1,11*]

1 Department of Medicinal Biotechnology, Graduate School of Pharmaceutical Sciences, Tokushima University, 1-78-1 Sho-machi, Tokushima 770-8505, Japan

2 Department of Pharmacology, Kawasaki Medical School, 577 Matsushima, Kurashiki, Okayama 701-0192, Japan

3 Institute of Agrobiological Sciences, National Agriculture and Food Research Organization, 1-2 Owashi, Tsukuba, Ibaraki 305-8634, Japan

4 Division of Biological Chemistry and Biologicals, National Institute of Health Sciences, 3-25-26 Tonomachi, Kawasaki-ku, Kawasaki, Kanagawa 210-9501, Japan

5 Carbohydrate Research & Development Department, Fushimi Pharmaceutical Co., Ltd. 307 Minato-machi, Marugame, Kagawa 763-0042, Japan

6 Primate Research Institute, Kyoto University (PRI), 41-2 Kanrin, Inuyama, Aichi 484-8506, Japan
Current address: Center for the Evolutionary Origins of Human Behavior, Kyoto University (EHUB), 41-2 Kanrin, Inuyama, Aichi 484-8506, Japan

7 Department of Medical Neuroscience, Graduate School of Medical Sciences, Kanazawa University, 13-1 Kanazawa, Ishikawa 920-8640, Japan

8 Graduate School of Information Science, University of Hyogo, 7-1-28 Minatojimaminamimachi, Kobe Chuo-ku, Hyogo 650-0047, Japan

9 The Exploratory Research Center on Life and Living Systems, National Institutes of Natural Sciences, 5-1 Higashiyama, Myodaiji, Okazaki, Aichi 444-8787, Japan

10 Joint Department of Veterinary Medicine, Faculty of Applied Biological Sciences, Gifu University, 1-1 Yanagido, Gifu 501-1193, Japan

11 Department of Medicinal Biotechnology, Faculty of Pharmaceutical Sciences, Tokushima University, 1-78-1 Sho-machi, Tokushima 770-8505, Japan

12 Laboratory of Biochemistry, Kobe Pharmaceutical University, 4-19-1 Motoyama-kitamachi, Nada-ku, Kobe, Hyogo 658-8558, Japan

13 Medical Education Development Center, Gifu University, 1-1 Yanagido, Gifu 501-1194, Japan

#These authors contributed equally to this work: Chika Shinoda and Keisuke Kitakaze
*These authors jointly supervised this work: Takao Oishi and Kohji Itoh

*Corresponding authors:

Takao Oishi, Ph.D.

Center for the Evolutionary Origins of Human Behavior, Kyoto University (EHUB)

41-2 Kanrin, Inuyama, Aichi 484-8506, Japan

Phone: +81-568-63-0573

E-mail: oishi.takao.5e@kyoto-u.ac.jp

Kohji Itoh, Ph.D.

Professor Emeritus, Tokushima University

Department of Medicinal Biotechnology, Graduate School of Pharmaceutical Science, Tokushima University

1-78-1 Shoumachi, Tokushima 770-8505, Japan

Phone: +81-90-4977-0234

E-mail: kitoh@tokushima-u.ac.jp

Gene	Protein	Disease
ARSB	arylsulfatase B	MPS VI (Maroteaux-Lamy syndrome)
CTSA	cathepsin A	galactosialidosis
ECM1	extracellular matrix protein 1	Urbach–Wiethe disease
GALNS	galactosamine (*N*-acetyl)-6-sulfatase	MPS IV-A (Morquio A syndrome)
GLB1	galactosidase beta 1	MPS IV-B (Morquio B syndrome)
GLB1	galactosidase beta 1	GM1 gangliosidosis
GNPTAB	*N*-acetylglucosamine-1-phosphate transferase subunits alpha and beta	mucolipidosis II/III alpha/beta
GNPTG	*N*-acetylglucosamine-1-phosphate transferase subunit gamma	mucolipidosis III gamma
GNS	glucosamine (*N*-acetyl)-6-sulfatase	MPS III-D (Sanfilippo D syndrome)
GUSB	glucuronidase beta	MPS VII (Sly syndrome)
HEXA	hexosaminidase subunit alpha	GM2 gangliosidosis type B (Tay-Sachs disease)
HEXB	hexosaminidase subunit beta	GM2 gangliosidosis type O (Sandhoff disease)
HGSNAT	heparan-alpha-glucosaminide *N*-acetyltransferase	MPS III-C (Sanfilippo C syndrome)
HYAL1	hyaluronidase 1	MPS IX (Natowicz syndrome)
IDS	iduronate 2-sulfatase	MPS II (Hunter syndrome)
IDUA	alpha-L-iduronidase	MPS I (Hurler/Hurler-Scheie/Scheie syndrome)
MCOLN1	mucolipin TRP cation channel 1	mucolipidosis type IV
NAGA	alpha-*N*-acetylgalactosaminidase	Schindler disease; Kanzaki disease
NAGLU	*N*-acetyl-alpha-glucosaminidase	MPS III-B (Sanfilippo B syndrome)
NEU1	neuraminidase 1	sialidosis
SGSH	*N*-sulfoglucosamine sulfohydrolase	MPS III-A (Sanfilippo A syndrome)
SUMF1	sulfatase modifying factor 1	multiple sulfatase deficiency

Supplementary Table 1. Verification of the presence or absence of mutations in the responsible gene for lysosomal diseases.

	nmol per mg creatinine (mol %)								
	MPS I #2			MPS I #3			WT		
	Hepase	HSase	Hepase HSase	Hepase	HSase	Hepase HSase	Hepase	HSase	Hepase HSase
ΔDiHS-0S	0.2 (1.4%)	3.7 (26.1%)	3.8 (24.4%)	ND	53.3 (39.7%)	25.8 (28.7%)	ND	ND	ND
ΔDiHS-6S	4.2 (28.6%)	0.9 (6.3%)	0.6 (3.8%)	ND	ND	ND	ND	ND	ND
ΔDiHS-NS	4.3 (29.2%)	6.6 (46.5%)	8.4 (53.9%)	57.2 (79.3%)	49.8 (37.2%)	48.4 (53.7%)	1.0 (100%)	1.0 (100%)	1.2 (100%)
ΔDiHS-diS$_1$	ND	ND	ND	ND	ND	ND	ND	ND	ND
ΔDiHS-diS$_2$	4.0 (27.2%)	3.0 (21.1%)	2.8 (17.9%)	14.9 (20.7%)	30.9 (23.1%)	15.8 (17.6%)	ND	ND	ND
ΔDiHS-triS	2.0 (13.6%)	ND	ND	ND	ND	ND	ND	ND	ND
Total	14.7	14.2	15.6	72.1	134.0	90.0	1.0	1.0	1.2

Supplementary Table 2. Disaccharide composition of urinary HS/Hep chains from WT and MPS I macaques. Hepase: heparinase, HSase: heparitinase, ΔDiHS-0S: ΔHexAα1-4GlcNAc, ΔDiHS-6S: ΔHexAα1-4GlcNAc(6-*O*-Sulfate), ΔDiHS-NS: ΔHexAα1-4GlcN(*N*-Sulfate), ΔDiHS-diS$_1$: ΔHexAα1-4GlcN(*N*-, 6-*O*-Sulfate), ΔDiHS-diS$_2$: ΔHexA(2-*O*-Sulfate)α1-4GlcN(*N*-Sulfate), ΔDiHS-triS: ΔHexA(2-*O*-Sulfate)α1-4GlcN(*N*-, 6-*O*-Sulfate), ND: not detected.

	nmol per mg creatinine (mol %)								
	MPS I #2			MPS I #3			WT		
	Chase ABC	Chase AC-II	Chase B	Chase ABC	Chase AC-II	Chase B	Chase ABC	Chase AC-II	Chase B
ΔDi-0S	1.3 (9.6%)	1.8 (10.6%)	0.3 (2.5%)	ND	14.4 (17.6%)	10 (19.0%)	ND	0.1 (1.0%)	0.1 (5.3%)
ΔDi-6S	0.5 (3.7%)	1.8 (10.6%)	1.3 (10.9%)	69.8 (73.9%)	60.5 (74.2%)	6.5 (12.4%)	1.7 (16.2%)	1.3 (12.5%)	0.5 (26.3%)
ΔDi-4S	11.8 (86.7%)	13.4 (78.8%)	10.3 (86.6%)	24.7 (26.1%)	6.7 (8.2%)	36 (68.6%)	8.8 (83.8%)	9.0 (86.5%)	1.3 (68.4%)
Total	13.6	17.0	11.9	94.5	81.6	52.5	10.5	10.4	1.9

Supplementary Table 3. Disaccharide composition of urinary CS/DS chains from WT and MPS I macaques. Chase: chondroitinase, ΔDi-0S: ΔHexAβ1-3GalNAc, ΔDi-6S: ΔHexAβ1-3GalNAc(6-*O*-Sulfate), ΔDi-4S: ΔHexAβ1-3GalNAc(4-*O*-Sulfate), ND: not detected.

Supplementary Table 4. Hematological and blood chemistry data in the MPS I macaque following ERT

Variable	Abbreviation	Units	1	135	149	162	177	191	205	219	282	379	392	406	421	**Day**
			KUPRI	KUPRI	KUPRI	KUPRI	KUPRI	KUPRI	KUPRI	KUPRI	KUPRI	AMT	AMT	AMT	AMT	**Institutions**
Glucose	GLU	mg/dL	138	89	111	80	91	85	94	62	127	80	126	87	100	
Blood urea nitrogen	BUN	mg/dL	14.9	15	14.9	16.6	12.7	11.4	10.2	13.5	12.2	15.1	14.5	13.5	15.3	
Creatinine	CRE	mg/dL	0.4	0.5	0.4	0.4	0.4	0.4	0.4	0.4	0.4	0.48	0.51	0.5	0.42	
Total cholesterol	TCHO	mg/dL	162									125	125	114	119	
Triglycerides	TG	mg/dL	51									72	40	29	54	
Total bilirubin	TBIL	mg/dL	0.1	0.1	0.1	0.3	0.1	0.2	0.2	0.2	0.2	<0.1	0.1	0.1	0.1	
Calcium	Ca	mg/dL	7.1	6.1	6.4	6.8	5.9	6	6.6	7	6.9	9	8.1	8.2	8	
Phosphate	IP	mg/dL	3.6	3.6	3.2	4.1	3.1	2.8	2.9	3.6	3.4	3.3	3.7	3.5	2.6	
Total protein	TP	g/dL	6.1	6	5.9	6.4	5.5	5.6	6	6.3	5.7	6.9	6.6	6.6	6.3	
Albumin	ALB	g/dL	3	2.9	3.1	3.4	2.6	2.6	3	3.2	3.2	3.7	3.3	3.4	3.2	
Aspartate aminotransferase	AST (GOT)	IU/L	19	23	33	38	27	24	23	22	22	21	28	29	25	
Alanine aminotransferase	ALT (GPT)	IU/L	35	66	148	148	79	68	45	37	31	38	71	81	65	
Creatinine phosphokinase	CPK	IU/L	382	435	132	234	120	619	49	197	239	277	329	206	230	
Sodium	Na	mEq/L	150	148	148	147	150	151	151	145	146	139	141	141	142	
Potassium	K	mEq/L	2.9	2.9	3.2	3.1	2.7	2.7	3.6	3.1	2.8	4.7	3.3	3.4	3.2	
Chloride	Cl	mEq/L	110	111	107	107	113	113	111	106	108	102	105	106	111	
Gamma glutamyl transpeptidase	γGTP	IU/L	106	132	182	214	149	127	68	77	84	116	167	205	147	
Alkaline phosphatase	ALP	IU/L	736	534	541	441	313	394	319	377	616					
White blood cell	WBC	$\times 10^3$/μL	7900	9200	7400	7300	6700	6300	8700	9100	6400	8200	8250	7450	6760	
Red blood cell	RBC	$\times 10^6$/μL	504	472	463	463	410	442	463	457	476	479	448	457	440	
Hemoglobin	Hb	g/dL	12.4	12	11.7	11.6	10.4	11.2	11.7	11.4	11.7	12.7	12	12.4	12	
Hematocrit	Ht	%	38.2	33.6	35.6	35.8	32.4	34.8	36	35.7	36.9	41.8	38.3	39.5	38.1	
Mean corpuscular volume	MCV	fL	75.8	71.2	76.9	77.3	79	78.7	77.8	78.1	77.5	87.2	85.4	86.3	86.5	
Mean corpuscular hemoglobin	MCH	pg	24.6	25.4	25.3	25.1	25.4	25.3	25.3	24.9	24.6	27.9	27.3	27.1	27.6	
Mean corpuscular hemoglobin concentration	MCHC	%	32.5	35.7	32.9	32.4	32.1	32.2	32.5	31.9	31.7	32	31.9	31.4	31.9	
Platelet	PLT	$\times 10^5$/μL	25.2	27.3	21	21.2	17.7	21.6	22.3	22	23.7	21.6	21.3	27.8	22.8	
WBC-small cell ratio	W-SCR	%	82		67	68.2	65.2	71.1	55.3	49.5	74.6					
WBC-middle cell ratio	W-MCR	%	1.9		2.3	3.9	3	2.7	2.9	2.8	2.4					
WBC-large cell ratio	W-LCR	%	16.1		30.7	27.9	31.8	26.2	41.8	47.7	23					
WBC-small cell count	W-SCC	$\times 10^3$/μL	65		50	50	44	45	48	45	48					
WBC-middle cell count	W-MCC	$\times 10^3$/μL	2		2	3	2	2	3	3	2					
WBC-large cell count	W-LCC	$\times 10^3$/μL	12		22	20	21	16	36	43	14					
RBC distribution width-standard deviation	RDW-SD	fL	35.5	37.5	37	38	38	37.6	35.5	37	36.3					
RBC distribution width-coefficient of variation	RDW-CV	%	11.7	11.8	12.1	12.5	12.7	12.1	11.5	12.1	11.8					
Platelet distribution width	PDW	fL	15.3	11.8	12.1	13.6	11.8	11.4	11.6	13.6	12.9					
Mean platelet volume	MPV	fL	12.5	11	10.6	12	10.7	10.2	10.4	11.8	11					
Platelet-large cell ratio	P-LCR	%	49.4	32	28.7	42.3	28	26.1	27.6	39	32.7					
C-reactive protein	CRP	mg/dL	0.3	0.3	0.3	0.3	0.3	0.3	0.3	0.3	0.3	0.58	<0.01	0.02	<0.01	
Lipase	LIP	IU/L										60	45	37	38	
Alkaline phosphatase isozymes	ALPi	IU/L										122	102	105	107	

KUPRI: Primate Research Institute, Kyoto University
AMT: Animal Medical Technology (Nagoya, Japan)

Mannose
GlcNAc
Glucose
Fucose

	Asn 110	Asn 190	Asn 336	Asn 372	Asn 415	Asn 451
M9Glc2				3.0		
M9Glc				9.8		
M9	0.1			31.3		
M8	1.1			7.8		
M7	5.6			24.5		
M6	5.1	1.5		14.0		
M5	9.2	0.8		8.5	35.3	
M4	1.1					
M3	4.4	8.6	1.3		41.3	
FM3	0.5	3.0				0.9
M2	0.9	3.1	7.9			7.7
A2		14.1	10.1			3.4
FA2		3.2	0.5			
A1	13.9	18.3	5.4		23.4	3.4
FA1	1.2	4.8	1.3			
M4A1	4.1					
M5A1	2.0					
GlcNAc	50.7	40.8	67.9			84.5
GlcNAc+F			5.2			
(–)		1.6	0.4	1.0		

Supplementary Figure 1. *N*-glycan profiles of GlcNAc-IDUA. *N*-glycan compositions and percentage abundances of GlcNAc-IDUA. Glucose (blue circle), GlcNAc (blue square), mannose (green circle), and fucose (red triangle). M9Glc2, $Glc_2Man_9GlcNAc_2$; M9Glc, $GlcMan_9GlcNAc_2$; M9, $Man_9GlcNAc_2$; M8, $Man_8GlcNAc_2$; M7, $Man_7GlcNAc_2$; M6, $Man_6GlcNAc_2$; M5, $Man_5GlcNAc_2$; M4, $Man_4GlcNAc_2$; M3, $Man_3GlcNAc_2$; FM3, $Man_3GlcNAc_2Fuc$; M2, $Man_2GlcNAc_2$; A2, $GlcNAc_2Man_3GlcNAc_2$; FA2, $GlcNAc_2Man_3GlcNAc_2Fuc$; A1, $GlcNAcMan_3GlcNAc_2$; FA1, $GlcNAcMan_3GlcNAc_2Fuc$; M4A1, $GlcNAcMan_4GlcNAc_2$; M5A1, $GlcNAcMan_5GlcNAc_2$; GlcNAc+F, GlcNAcFuc.

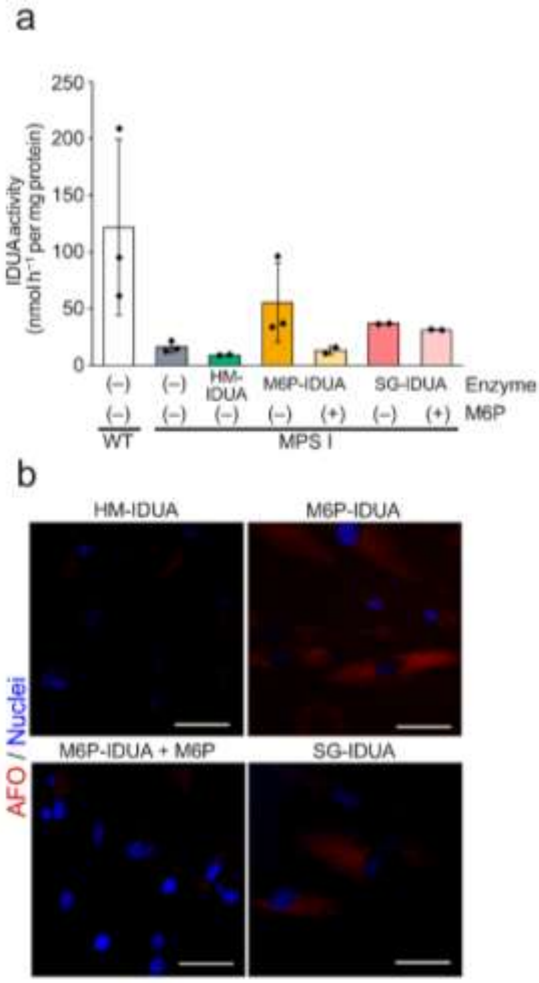

Supplementary Figure 2. Effect of enzyme replacement with hIDUAs on MPS I macaque-derived fibroblasts. (a) Intracellular IDUA activity in MPS I fibroblasts after treatment with M6P- and SG-IDUA. Before enzyme treatment, fibroblasts were treated (+) or not treated (–) with M6P. Error bars show mean ± SD (n = 2–3). **(b)** IDUA delivery imaging. AFO-labeled HM-, M6P-, and SG-IDUA were added to MPS I fibroblasts. Blue, nuclei; red, AFO. Scale bars: 50 μm.

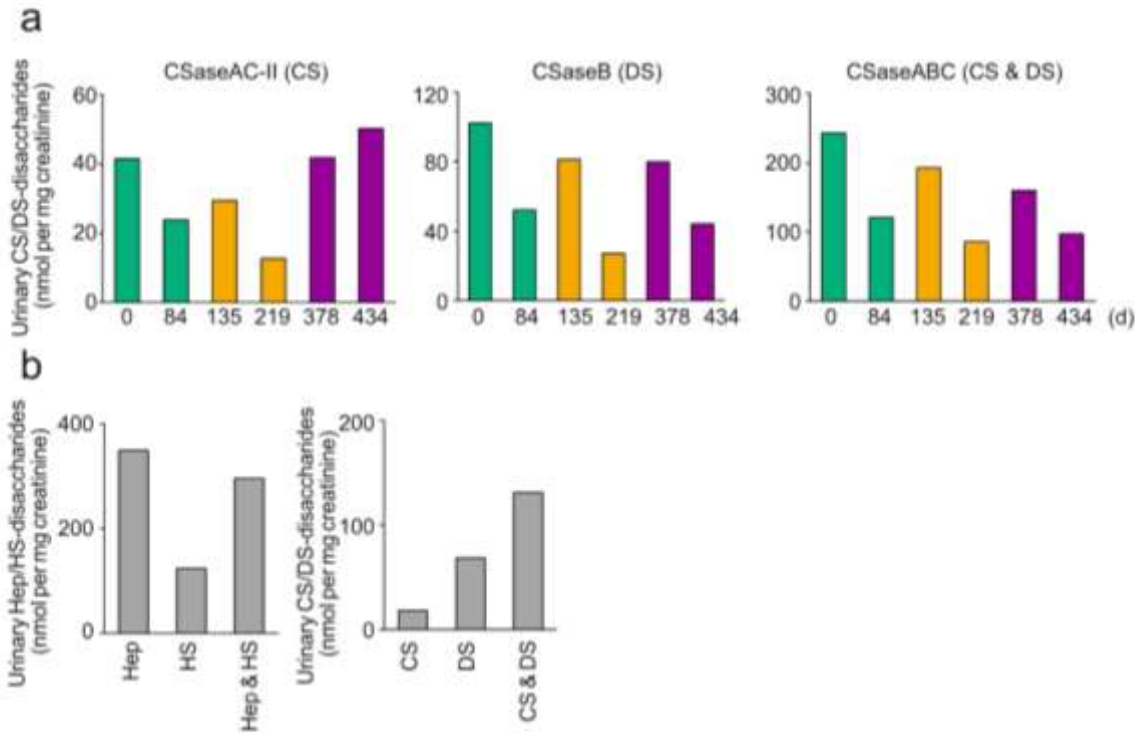

Supplementary Figure 3. Urinary GAG levels in the enzyme-treated MPS I macaque. (a) Quantification of urinary CS and DS levels in the MPS I macaque. **(b)** Quantification of urinary GAG levels in the MPS I macaque 2 years after completion of ERT. MPS I (#2): female, 8 years 4 months old. Hep: heparin, HS: heparan sulfate, CS: chondroitin sulfate, DS: dermatan sulfate.

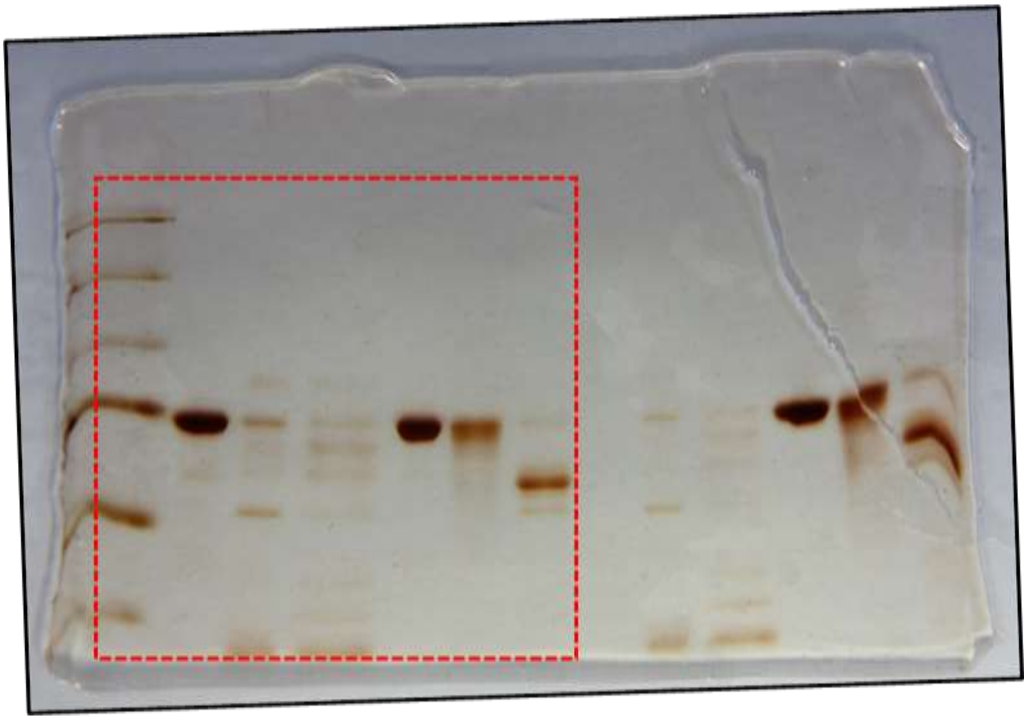

Supplementary Figure 4. Uncropped gel for Fig. 1b.

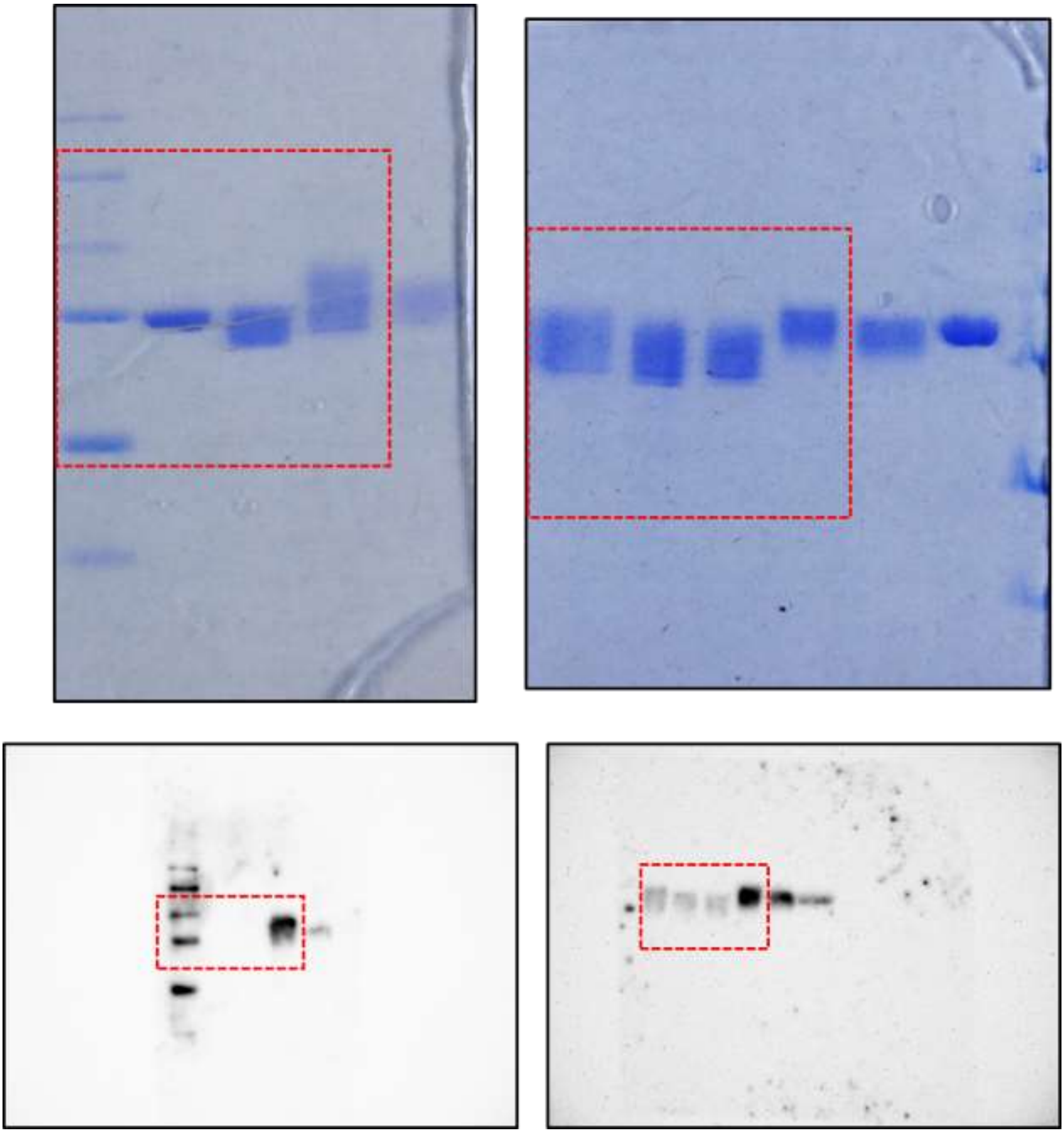

Supplementary Figure 5. Uncropped gel and blotting membrane for Fig. 2b, c, d and e.

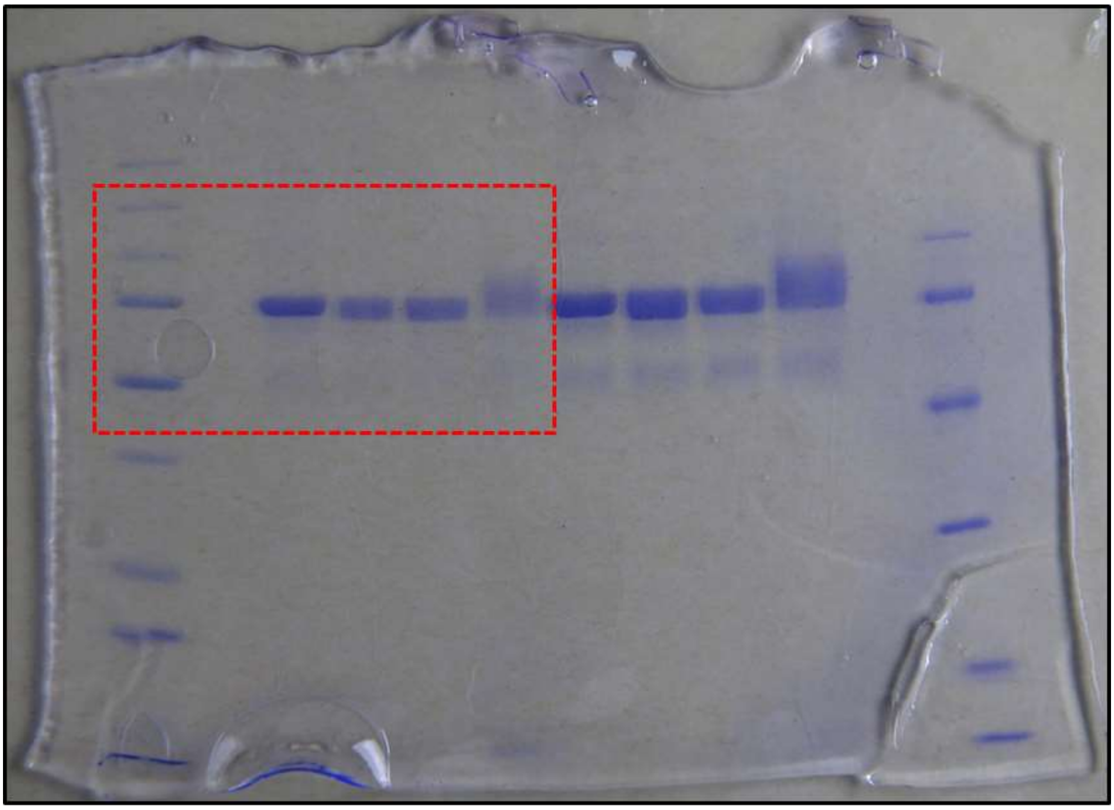

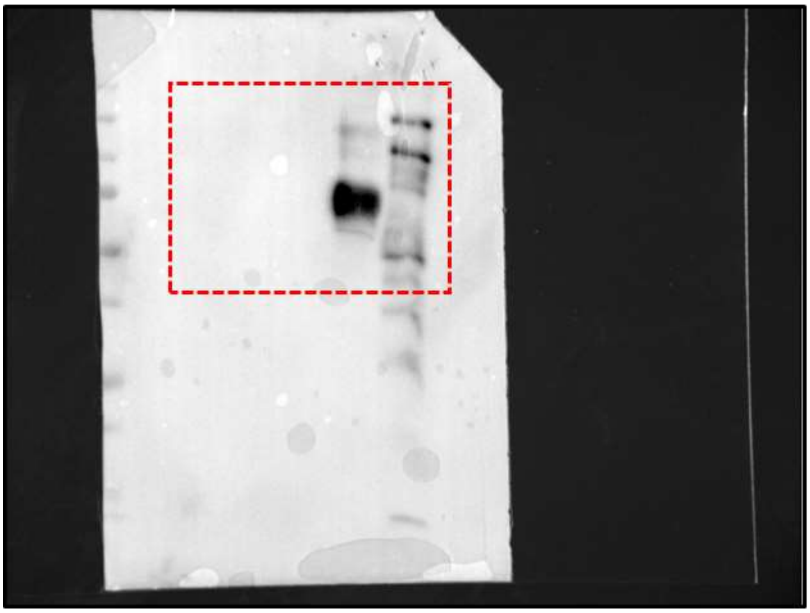

Supplementary Figure 6. Uncropped gel and blotting membrane for Fig. 3b and c.
